# Endoscopic Management Options for Gastroesophageal Reflux Disease

**DOI:** 10.7759/cureus.62069

**Published:** 2024-06-10

**Authors:** Bhavana Sreepad, Karteek Chennupati, Muhammad Shehroz Zeeshan, Zeeshan Ramzan

**Affiliations:** 1 Medical School, TCU Burnett School of Medicine, Fort Worth, USA; 2 Gastroenterology, Texas Health Harris Methodist Hospital, Fort Worth, USA; 3 Gastroenterology, TCU Burnett School of Medicine, Fort Worth, USA

**Keywords:** concomitant transoral incisionless fundoplication (ctif), transoral incisionless fundoplication (tif), stretta, endoscopic treatment options, gastroesophageal reflux disease (gerd)

## Abstract

Gastroesophageal reflux disease (GERD) is a prevalent condition that affects a significant portion of the Western population. Despite its benign pathophysiology, it has the potential to cause serious complications over time, ranging from conditions that are benign, premalignant, and/or malignant. Traditional treatment options include lifestyle measures, anti-secretory medications (e.g., proton pump inhibitor (PPI)), and surgical options (e.g., Nissen and Toupet fundoplication). However, recent studies have revealed long-term side effects of anti-secretory medications. Moreover, surgical options, though effective, are considered invasive and associated with potential complications. In the current age of ongoing research in minimally invasive options, endoscopic treatment of GERD has become popular. As a result, procedures such as radiofrequency treatment and transoral incisionless fundoplication (TIF) have gained FDA approval and are currently being covered by most insurance. In this review article, we will discuss pre-procedural workup, appropriate patient selection, advantages, disadvantages, procedure techniques, and follow-up of patients who undergo various endoscopic treatments for GERD. In addition, we will review the short and long-term success of these techniques in improving quality of life, use of PPI, and improvement in symptoms considering published data in high-quality peer-reviewed journals.

## Introduction and background

Gastroesophageal reflux disease (GERD) is one of the most commonly presenting gastrointestinal (GI) diseases today and is defined as the pathological retrograde movement of gastric contents into the esophagus due to inadequate pressure gradients [1]. Symptoms of GERD include heartburn, chest pain, dysphagia, odynophagia, and cough. Normally, the lower esophageal sphincter (LES) acts as an anti-reflux barrier to prevent this retrograde flow. However, if the LES becomes incompetent or there is an increase in intra-abdominal pressure, gastric contents can reflux into the esophagus. This can induce metaplastic changes to squamous epithelium that can eventually progress to Barrett’s esophagus and then carcinoma.

Initial treatment for GERD involves lifestyle and dietary changes, as well as medications, including proton pump inhibitors (PPIs) and histamine-2 receptor antagonists. While such medications have allowed many patients to experience symptom relief, they are not a one-size-fits-all therapeutic option. Some patients may not tolerate medications or may not adequately respond well to such therapy. Refractory GERD (rGERD) is defined as persistent anti-reflux symptoms despite such first-line management and PPI treatment for at least eight weeks.

Despite the high incidence of rGERD, the nuances in patient presentation and the multifaceted nature of the disease have proved challenging in providing adequate treatment options for every patient. Patients with rGERD have conventionally been treated with laparoscopic anti-reflux surgery (LARS), which is not only invasive but also associated with symptoms such as flatulence and gas-bloat syndrome [2].

Endoscopic management of GERD has expanded the therapeutic options available to patients and is considered a promising alternative to the surgical approach. The purpose of this review article is to present endoscopic treatment options for rGERD in the context of recent research, appropriate patient selection, and the advantages and disadvantages of each treatment.

## Review

Radiofrequency treatment for GERD 

**Figure 1:**
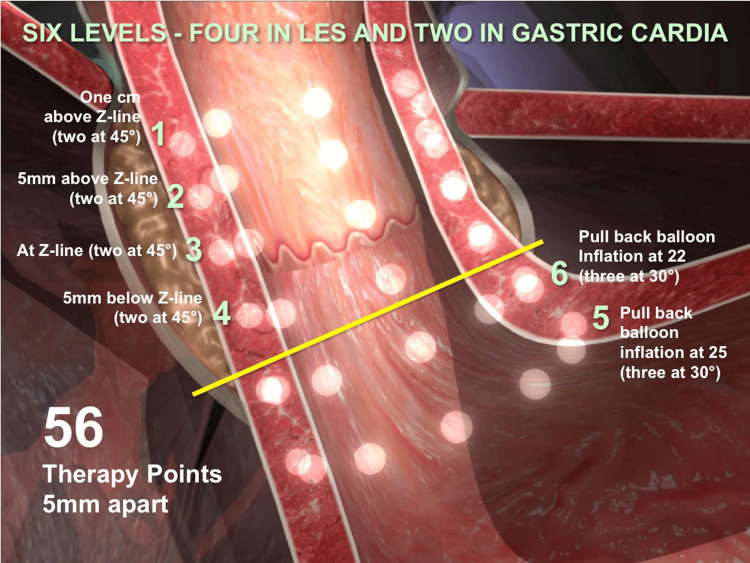
The procedure of Stretta involves the targeted application of radiofrequency thermal energy and specific points as detailed in this figure. © 2024 Restech | Mederi-RF, LLC Credit: Restech is the sole owner of the attached image(s) and hereby authorizes the use of these images in the manuscript for the Cureus issue provisionally entitled "Endoscopic Management Options for Gastroesophageal Reflux Disease," print and electronic 2024.

An important minimally invasive innovation to treat gastroesophageal reflux includes the application of radiofrequency energy to the LES complex. This procedure is commonly known as Stretta, which is an Italian word meaning "tight." The technique involves introducing a catheter system in the distal esophagus and gastric cardia in a stepwise fashion to deliver radiofrequency thermal energy (465 kHz, 2-5 watts per channel, 80 volts maximum at 100 to 800 ohms; see Figure 1). A target temperature of 85 °C is achieved by delivering radiofrequency energy via each electrode for 60 seconds.

**Figure 2:**
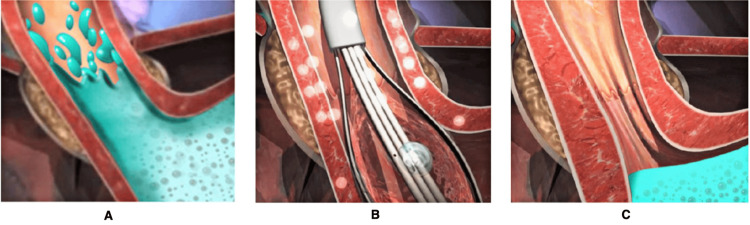
Mechanism of action of Stretta. Using a catheter, a balloon is inflated at the distal esophagus followed by the application of radiofrequency in a stepwise fashion. Post Stretta there is the tightening of the gastroesophageal junction. © 2024 Restech | Mederi-RF, LLC A) Reflux: a weak muscle that allows stomach contents to reflux into the esophagus; B) Stretta therapy: treats the muscle with radiofrequency energy; C) post Stretta: the regenerated, thicker muscle prevents reflux. Credit: Restech is the sole owner of the attached image(s) and hereby authorizes the use of these images in the manuscript for the Cureus issue provisionally entitled "Endoscopic Management Options for Gastroesophageal Reflux Disease," print and electronic 2024.

Several theories on the potential pathophysiology of how Stretta works have been postulated. This includes collagen contraction, submucosal fibrosis, and muscle hypertrophy due to the thermal effect of the Stretta catheter (see Figure 2). This results in decreased compliance, decreased esophageal acid sensitivity, and reduced postprandial transient LES relaxation.

Ideal candidates for Stretta are patients who have pathological GERD manifested by typical symptoms and abnormal acid reflux testing. Patients who wish to be off long-term PPI medications and prefer the least invasive option would be suitable candidates for Stretta. Poor candidates for Stretta include patients with large hiatal hernia (greater than 2 cm), significant dysphagia (with evidence of spastic motility disorder on esophageal manometry), and severe esophagitis [3].

Inclusion and Exclusion Criteria for Stretta

The inclusion criteria encompass patients undergoing treatment for GERD with or without hiatal hernia, those experiencing recurrent reflux symptoms post-fundoplication, patients with GERD symptoms following sleeve gastrectomy or upper GI surgery, those requiring bridging therapy for reflux control before fundoplication, and patients for whom more invasive procedures like laparoscopic fundoplication or transoral incisionless fundoplication (TIF) are contraindicated, technically challenging, or too invasive. Additionally, patients wishing to stop PPI in favor of a less invasive endoscopic option are included. The exclusion criteria comprise patients with known absolute contraindications, relative contraindications, those under 18, individuals without a GERD diagnosis, those suffering from severe esophagitis, dysphagia due to achalasia or incomplete LES relaxation, pregnant women, individuals with hiatal hernia greater than 2 cm, and poor surgical candidates classified as ASA IV.

Efficacy of Stretta

Numerous studies have examined the efficacy of radiofrequency treatment for GERD. Significant improvement in symptom profile and PPI use has been documented in short-term [4,5] and long-term studies [6]. However, no significant improvement in LES basal pressure was reported despite reduced esophageal acid exposure. Nevertheless, improved outcomes compared to PPI therapy alone suggest the utility of Stretta in patients who want to avoid surgery and potential side effects from long-term PPI use.

Significant advantages of using Stretta for the management of GERD are the ease of use, excellent safety profile, and cost-effectiveness. Patients who are considered poor surgical candidates such as significantly obese patients [7], patients with a history of prior gastric surgery [8], history of failed fundoplication [9] may be appropriate candidates for Stretta due to excellent safety profile. In a meta-analysis involving over 2400 patients, the rate of significant adverse events for Stretta was less than 1% [4]. Several studies have shown that it is a very cost-effective approach compared to other modalities (such as long-term PPI use and surgical fundoplication) [10,11].

TIF

TIF stands as a minimally invasive endoscopic solution for individuals with rGERD [12]. This procedure, first introduced in 2006 and subsequently evolved into TIF 2.0, has obtained approval since 2007. It involves the anatomical reconstruction of the valve through the folding of the gastric fundus, thereby reinstating the strength of the LES and the gastroesophageal flap valve (GEFV). TIF has demonstrated successful outcomes when utilized with medical devices like EsophyX (EndoGastric Solutions, Redmond, United States), offering a non-surgical alternative to patients dealing with rGERD. The current TIF 2.0 iteration proves to be less invasive compared to the traditional surgical method of laparoscopic Nissen Fundoplication (LNF), and it carries a reduced risk of side effects like dysphagia and flatulence [2].

Mechanism of Action

**Figure 3:**
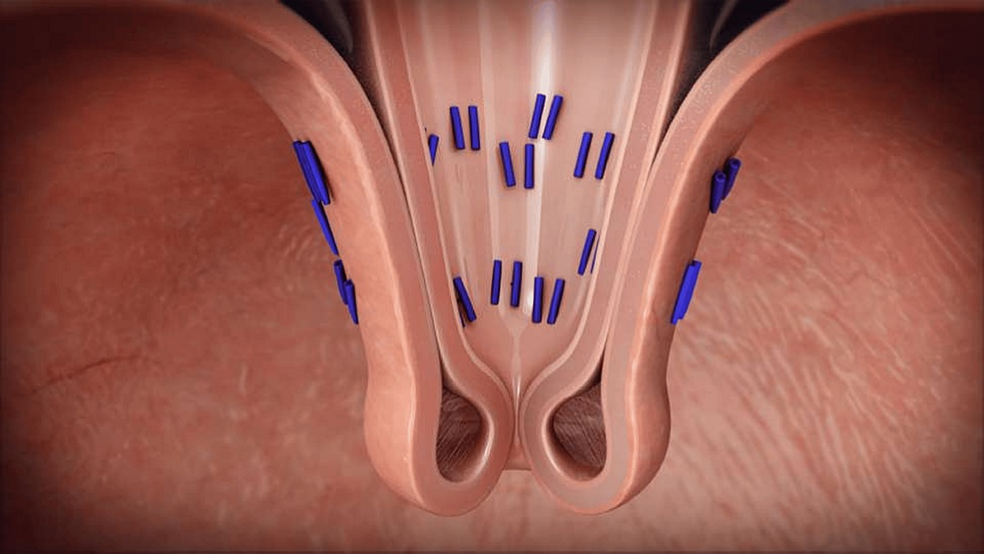
Depiction of the suture-like polypropylene H-fasteners used in the TIF procedure TIF: transoral incisionless fundoplication Credit: Courtesy of EndoGastric Solutions, Inc. We are pleased to grant The Cureus Journal of Medical Science non-exclusive rights to use the image shown below for inclusion in the article “Endoscopic Management Options for Gastroesophageal Reflux Disease” to be published in The Cureus Journal of Medical Science.

The TIF procedure is conducted under general anesthesia with the device inserted transorally through the mouth, providing clear visualization of the gastroesophageal junction (GEJ). Stomach walls are retracted into the endoscope, and suture-like polypropylene H-fasteners are implemented 2 to 3 cm above the GEJ in conjunction with a 270-degree esophagogastric wrap [13]. This sequence is reiterated multiple times using the EsophyX-Z device, establishing a high-pressure zone and a strong anti-reflux valve (Figure 3).

Remarkably, in 2016, the introduction of the EsophyX-Z device streamlined the TIF 2.0 procedure by enabling automated, accelerated fastener deployment similar to a surgical stapler, ensuring a more consistent and reliable application. Additionally, the EsophyX-Z device allows for the incorporation of an average of 20 or more fasteners, thereby enhancing the effectiveness of preventing GERD symptoms and preserving the integrity of the angle of His [14].

An exploratory study on the mechanism of action of TIF revealed a reduction in the occurrence of postprandial transient LES relaxations (TLESRs) and a decrease in esophagogastric junction (EGJ) distensibility following the TIF procedure [15]. As this process allows gas to escape from the stomach, it minimizes the occurrence of gas bloat as a side effect. The reduced frequency of TLESR improved EGJ distensibility and the lower incidence of gas-bloat compared to LNF positions TIF as a promising alternative for individuals with rGERD.

Patient Selection

Patient selection plays a central role in ensuring the safety and success of TIF as a treatment option. It is imperative that candidates have a compelling need for anti-reflux therapy, especially when standard approaches like medications and lifestyle modifications have proven insufficient in alleviating their symptoms. The assessment of candidate suitability often involves the application of the Hill criteria, a method used to ascertain the structural characteristics of the GEJ and the presence of hiatal hernias. Notably, a systematic review conducted by Osman et al. in 2021 revealed a significant correlation between an abnormal GEFV classification of Hill Grade III or IV and the presence of symptomatic GERD as well as erosive esophagitis [16].

Inclusion and Exclusion Criteria for TIF

The inclusion criteria for the study are patients with a BMI less than 35 kg/m², those with moderate to severe rGERD, patients with a hiatal hernia less than 2 cm, individuals aged 18 and older, patients who do not respond to PPI, those with Hill I or II classification, those able to adhere to a postoperative diet regimen, and individuals available for follow-up at the 6-month and 12-month marks. Additionally, patients must have signed informed consent and can have a hiatal hernia greater than 2 cm if laparoscopic repair reduces it to 2 cm or less. The exclusion criteria include patients with a BMI greater than 35 kg/m², those with portal hypertension and esophageal varices, esophageal strictures or stenosis, esophageal diverticula, achalasia, scleroderma, dermatomyositis, severe esophagitis, obstructions, or esophageal infections. Other exclusions are paraesophageal hiatal hernia, pregnancy, significant comorbidities, inability to adhere to a postoperative diet regimen, coagulation disorders, chronic cough unrelated to GERD, cervical spine fusion with neck mobility restrictions, gastroparesis, pyloric stenosis, diverticula, or any obstruction to the gastric outlet, and anatomical factors preventing the insertion of the EsophyX device.

The Hill criteria is a valuable tool employed to help determine the appropriateness of TIF for a given patient. In cases where individuals exhibit normal anatomical features according to the Hill criteria and their intermittent GERD symptoms are effectively managed with occasional use of anti-acid medications, the recommended approach typically involves intense lifestyle management alongside episodic use of anti-acid medication [14]. Lifestyle modification includes eating dinner 3-4 hours before bedtime, keeping head elevated 4-6 inches above the ground at nighttime, dietary modification to lose excess weight, avoiding wearing tight-fitted clothes, etc. Patients characterized as Hill Grade II typically present with an incompetent LES, as evidenced by a less prominent GEFV and respiration-dependent closure observed during endoscopy. Patients falling within the Hill Grade II category, without concomitant hiatal hernias, and presenting definitive signs of rGERD, are often deemed ideal candidates for the TIF procedure [14]. Conversely, when patients exhibit Hill Grade III, the GEFV is minimally present, and there is an absence of closure around the endoscope, distinguishing them from Grade II individuals. For those categorized as Hill Grade IV, the GEFV is no longer observable. Importantly, TIF alone is not designed to address the crural defect typically found in Hill Grade III or IV patients, rendering it less suitable [17]. In such cases, concomitant TIF (cTIF) involving hiatal hernia repair and TIF procedure has been identified as a safe and effective alternative approach [18].

In a recent article, the American Foregut Society (AFS) expands on this Hill classification by incorporating axial hiatal hernia length (L), hiatal aperture diameter (D), and presence or absence of the GEFV (F) [17]. The anti-reflex barrier is supported by the LES as a physiological barrier, the GEFV as a mechanical barrier, and the crural diaphragm as both. AFS assembled a group of 13 members to evaluate the effectiveness of the Hill grade criteria and identified several limiting factors [17]. These include the Hill grade’s over-emphasis on the flap valve, a subjective and ambiguous approach to hiatal hernias to the determination of a competent GEFV, and its overall variability among endoscopists [17]. While further research is needed to correlate this expanded criterion with severity prediction, it aims to address the limitations of the Hill Grade classification comprehensively, fostering standardization among providers.

cTIF

cTIF was approved by the FDA in 2017 and has been studied as a feasible intervention for patients with Hill Grade III or IV with a large hiatal hernia (HH) > 2 cm. cTIF involves laparoscopic HH repair followed by the TIF portion of the procedure. This minimally invasive approach offers the potential for shorter recovery times, reduced discomfort, and effective treatment of GERD with a significant hiatal hernia.

In one study, patients with confirmed GERD and a hiatal hernia of more than 2 cm who underwent cTIF between 2018 and 2020 were studied [18]. Symptoms before and after the procedure were assessed using questionnaires. Significant improvements were observed in symptom frequency and severity, along with enhancements in the patient's quality of life after cTIF [19]. Furthermore, the need for medication significantly decreased, and minimal gas bloat symptoms were reported, showcasing the procedure’s effectiveness [18]. A more recent study also found that the majority of its participants reported a 75% or higher satisfaction following cTIF, along with a decrease in GERD-related symptoms [20].

cTIF also involves effective collaboration between gastroenterologists and GI surgeons to optimize patient care and improve outcomes. The collaborative approach between gastroenterology and surgery was well-received by patients, further emphasizing the possibility of cTIF in addressing the multifaceted aspects of GERD management [18]. Gastroenterologists and GI surgeons have differing and nuanced insights into the procedure. As detailed by Nguyen et al., the gastroenterologists comprehend the procedure from a laparoscopic viewpoint and the GI surgeons approach it endoscopically [21]. The difference in perspective has led to a better understanding of the criteria required for the ideal anti-reflux valve and suggested modifications to the current fundoplication techniques. The omega flap valve used in the cTIF procedure is described to be a fully created GEFV with effective intraluminal pressure that successfully avoids dysphagia and gas bloat syndrome [21]. Therefore, the authors suggest that cTIF presents a promising strategy to expand the eligibility for GERD treatment and offers several advantages over traditional laparoscopic fundoplication, notably a reduced likelihood of gas bloat symptoms [18,21].

pH scores have also been utilized to test the effectiveness of cTIF as a novel therapeutic option. A retrospective study discovered that among 22 patients, pH scores normalized in 21 patients (95%) after hiatal repair followed by TIF [21]. The average pH scores significantly improved from 35.3 to 10.9 (p<0.001). Consequently, the study concluded that cTIF substantially enhanced patient outcomes and allowed for the normalization of pH exposure [21]. cTIF has also been determined to be a safe and effective alternative to LNF [22]. Research comparing patients who underwent HH repair with TIF with HH repair with LNF found that the two approaches showed no significant differences in the rates of discontinuing or reducing PPI use, dysphagia, esophagitis, disrupted wrap, and HH recurrence [22]. In addition, the TIF group also exhibited a lower rate of new or worsening bloat at the six-month mark [22]. Therefore, TIF and cTIF stand as safe and effective options for patients who meet the criteria [19,23].

Inclusion and Exclusion Criteria for cTIF

The inclusion criteria specify that patients with Hill Grade III or IV and the presence of a hiatal hernia greater than 2 cm that can be repaired prior to TIF are eligible. The exclusion criteria refer to the standard exclusions for TIF, except for hiatal hernia dimensions, and the standard inclusion criteria for TIF, except for the Hill classification and hernia size.

Efficacy of TIF

TIF offers a minimally invasive approach, potentially resulting in shorter recovery times and improved patient outcomes [24]. Research supports the effectiveness and safety of TIF. Studies have shown significant reductions in reflux symptom index (RSI) scores at 6- and 12-month follow-ups, decreased PPI usage, and high patient satisfaction rates post-TIF [24]. Cost-effectiveness analyses indicate that TIF 2.0 is a cost-effective intervention for GERD patients, particularly those with persistent symptoms despite low-dose PPI treatment [25].

TIF has been shown to be an effective treatment option for rGERD, despite limited long-term research. A comprehensive literature search by Testoni et al. used four major scientific databases until May 2020 to identify studies reporting outcomes of TIF with follow-up durations exceeding three years [26]. According to this study, TIF can sustain high patient satisfaction rates over the long term, resulting in significant reductions in PPI usage, and consistent improvement in GERD-related symptoms as well as quality of life for patients. While it is important to acknowledge certain limitations, including the absence of complete clinical assessments in some long-term studies and variations in patient-reported satisfaction, it has been demonstrated objectively that the procedure is effective based on the significant reduction in overall PPI consumption and consistent improvements in GERD quality of life scores [26]. This study provides further evidence that TIF is a viable long-term alternative for the specific subset of GERD patients considered in this study [26].

TIF has also been studied as a potential safe and effective rescue option after a failed laparoscopic fundoplication. Revising a failed laparoscopic fundoplication is associated with high risk and lower rates of success [27]. In a recent study, a retrospective analysis was conducted to determine the safety and feasibility of TIF 2.0 after failed laparoscopic Nissen or Toupet Fundoplication (TIFFF) [27]. The mean GERD health-related quality of life (GERD-HRQL) significantly improved (p=0.014), and the mean RSI decreased (p=0.046) post TIF [27]. Subsequently, the use of PPIs also subsequently decreased from 85% to 55% [27]. While no statistically significant differences in efficacy were observed between TIFFF and performing the revision surgically, fewer adverse effects and shorter procedure length times were noted with TIFFF compared to surgical revisions [27]. Therefore, TIF can be an effective and safe option for patients post-failed fundoplication.

Additional endoscopic options

In a difficult subset of the patient population with GERD, such as patients with altered surgical anatomy, poor surgical candidates, etc., various other modalities have been used with some success. Mucosal ablation of the GEJ with argon plasma coagulation (APC) resulting in fibrosis and scarring has provided limited benefit. However, following that with endoscopic full-thickness suturing with an Overstitch device (Apollo Endosurgery, Inc., Austin, United States) has provided better outcomes with more than 50% of patients able to reduce or discontinue the use of PPI after the procedure [28]. This technique, mucosal ablation and suturing of the EG junction (MASE) require the application of APC to GEJ/gastric cardia followed by endoscopic suturing along the lesser curvature of gastric cardia in antegrade endoscopic position [28].

Another endoscopic approach involving endoscopic mucosal resection of EG junction resulting in scarring and decreased gastroesophageal reflux over time has been described. This technique called the anti-reflux mucosectomy (ARMS) technique has been shown to be safe and efficacious with up to two-thirds of patients with improved symptoms and decreased use of PPI in several studies [29]. A study aimed to compare the efficacy of ARMS and Stretta for the treatment of GERD. Results from a six-month follow-up showed that both ARMS and Stretta were effective in improving symptoms and overall quality of life. While no significant differences were observed between the two in terms of GERD questionnaire scores, HRQL, PPI withdrawal rates, or PPI reduction rates, the study found that both ARMS and Stretta were acceptable for patients with GEFV grades II and III, while ARMS should be selected for patients with GEFV grade IV [30].

Additional modification of this method includes semi-circumferential mucosectomy followed by full-thickness plication of GEJ complex, called “resection and plication” or RAP [31]. Data on this technique is sparse but encouraging with a low side effect profile, high technical success, and improved GERD-HRQL scores [31]. However, in patients where endoscopic mucosal resection is not feasible (such as due to scarring from prior resection or ablation), mucosal ablation with APC followed by suturing of the EG junction (MASE) technique can be considered.

Antireflux band mucosectomy (ARBM) represents a novel and minimally invasive treatment option for rGERD, aiming to deliver more effective symptom relief to patients. Utilizing an adult gastroscope, the procedure begins with visualization of the LES and the stomach, followed by retroflexion of the scope to visualize the cardia and fundus. Bands are then applied to the angle of His around the EGJ using suction. Typically, four bands are employed to achieve effective constriction of the junction [32]. This band placement induces scarring and fibrosis, resulting in the narrowing of the EGJ. With an average procedure time ranging from 6 to 15 minutes, ARBM presents a promising alternative for patients with rGERD who are not candidates for surgical exploration. A study investigating the efficacy of ARBM demonstrated a reduction in DeMeester scores, acid exposure time, and eventual cessation of PPI therapy within four weeks post-treatment for patients [32].

## Conclusions

Endoscopic treatment options for rGERD are effective choices for specific patient populations and offer several advantages over conventional surgical LARS. Options such as TIF and Stretta are less invasive, result in reduced hospital stays, and have fewer associated side effects. GERD is a spectrum disorder, thus requiring a spectrum of treatment options to address patient needs. Endoscopic treatment options accommodate this spectrum and, depending on the patient, can be part of an optimized treatment plan. The decision to propose an endoscopic treatment option requires careful consideration of the patient's symptoms, endoscopic findings, and prior interventions. While it has its benefits, these may not apply to every patient. Moreover, more research is needed to understand the long-term efficacy, side effect profile, and specific criteria for the different endoscopic treatments. Current research has shown that such interventions address nuanced patient needs and therefore should be strongly considered in patient care.

## References

[1] El-Serag HB, Sweet S, Winchester CC, Dent J (2014). Update on the epidemiology of gastro-oesophageal reflux disease: a systematic review. Gut.

[2] Kahrilas PJ, Boeckxstaens G, Smout AJ (2013). Management of the patient with incomplete response to PPI therapy. Best Pract Res Clin Gastroenterol.

[3] Jung HY (2021). In which situation is endoscopic radiofrequency anti-reflux therapy (Stretta) effective for controlling gastroesophageal reflux symptoms?. Clin Endosc.

[4] Fass R, Cahn F, Scotti DJ, Gregory DA (2017). Systematic review and meta-analysis of controlled and prospective cohort efficacy studies of endoscopic radiofrequency for treatment of gastroesophageal reflux disease. Surg Endosc.

[5] Slater BJ, Collings A, Dirks R (2023). Multi-society consensus conference and guideline on the treatment of gastroesophageal reflux disease (GERD). Surg Endosc.

[6] Viswanath Y, Maguire N, Obuobi RB, Dhar A, Punnoose S (2019). Endoscopic day case antireflux radiofrequency (Stretta) therapy improves quality of life and reduce proton pump inhibitor (PPI) dependency in patients with gastro-oesophageal reflux disease: a prospective study from a UK tertiary centre. Frontline Gastroenterol.

[7] Go MR, Dundon JM, Karlowicz DJ, Domingo CB, Muscarella P, Melvin WS (2004). Delivery of radiofrequency energy to the lower esophageal sphincter improves symptoms of gastroesophageal reflux. Surgery.

[8] Nevins EJ, Dixon JE, Viswanath YK (2021). The outcome of endoscopic radiofrequency anti-reflux therapy (Stretta) for gastroesophageal reflux disease in patients with previous gastric surgery: a prospective cohort study. Clin Endosc.

[9] Noar M, Squires P, Khan S (2017). Radiofrequency energy delivery to the lower esophageal sphincter improves gastroesophageal reflux patient-reported outcomes in failed laparoscopic Nissen fundoplication cohort. Surg Endosc.

[10] Comay D, Adam V, da Silveira EB, Kennedy W, Mayrand S, Barkun AN (2008). The Stretta procedure versus proton pump inhibitors and laparoscopic Nissen fundoplication in the management of gastroesophageal reflux disease: a cost-effectiveness analysis. Can J Gastroenterol.

[11] Funk LM, Zhang JY, Drosdeck JM, Melvin WS, Walker JP, Perry KA (2015). Long-term cost-effectiveness of medical, endoscopic and surgical management of gastroesophageal reflux disease. Surgery.

[12] McCarty TR, Itidiare M, Njei B, Rustagi T (2018). Efficacy of transoral incisionless fundoplication for refractory gastroesophageal reflux disease: a systematic review and meta-analysis. Endoscopy.

[13] Lee DP, Chang KJ (2022). Endoscopic management of GERD. Dig Dis Sci.

[14] Bazerbachi F, Krishnan K, Abu Dayyeh BK (2019). Endoscopic GERD therapy: a primer for the transoral incisionless fundoplication procedure. Gastrointest Endosc.

[15] Rinsma NF, Smeets FG, Bruls DW, Kessing BF, Bouvy ND, Masclee AA, Conchillo JM (2014). Effect of transoral incisionless fundoplication on reflux mechanisms. Surg Endosc.

[16] Osman A, Albashir MM, Nandipati K, Walters RW, Chandra S (2021). Esophagogastric junction morphology on Hill's classification predicts gastroesophageal reflux with good accuracy and consistency. Dig Dis Sci.

[17] Nguyen NT, Thosani NC, Canto MI (2022). The American foregut society white paper on the endoscopic classification of esophagogastric junction integrity. Foregut.

[18] Choi AY, Roccato MK, Samarasena JB (2021). Novel interdisciplinary approach to GERD: concomitant laparoscopic hiatal hernia repair with transoral incisionless fundoplication. J Am Coll Surg.

[19] Roy-Shapira A, Bapaye A, Date S, Pujari R, Dorwat S (2015). Trans-oral anterior fundoplication: 5-year follow-up of pilot study. Surg Endosc.

[20] Gisi C, Wang K, Khan F (2021). Efficacy and patient satisfaction of single-session transoral incisionless fundoplication and laparoscopic hernia repair. Surg Endosc.

[21] Nguyen NT, Chinn J, Chang K (2021). Collaboration between GI surgery & gastroenterology improves understanding of the optimal antireflux valve - the omega flap valve. Surg Endosc.

[22] Jaruvongvanich VK, Matar R, Reisenauer J (2023). Hiatal hernia repair with transoral incisionless fundoplication versus Nissen fundoplication for gastroesophageal reflux disease: a retrospective study. Endosc Int Open.

[23] Ajmera K, Thaimuriyil N, Shah N (2022). Recent advances in the endoscopic management of gastro-esophageal reflux disorder: a review of literature. Cureus.

[24] Haseeb M, Brown JR, Hayat U, Bay C, Bain PA, Jirapinyo P, Thompson CC (2023). Impact of second-generation transoral incisionless fundoplication on atypical GERD symptoms: a systematic review and meta-analysis. Gastrointest Endosc.

[25] McCarty TR, Jirapinyo P, James LP, Gupta S, Chan WW, Thompson CC (2022). Transoral incisionless fundoplication is cost-effective for treatment of gastroesophageal reflux disease. Endosc Int Open.

[26] Testoni S, Hassan C, Mazzoleni G (2021). Long-term outcomes of transoral incisionless fundoplication for gastro-esophageal reflux disease: systematic-review and meta-analysis. Endosc Int Open.

[27] Ghosh G, Choi AY, Dbouk M (2023). Transoral incisionless fundoplication for recurrent symptoms after laparoscopic fundoplication. Surg Endosc.

[28] Fortinsky KJ, Shimizu T, Chin M (2018). Mucosal ablation and suturing at the esophagogastric junction (MASE): a novel procedure for the management of patients with gastroesophageal reflux disease. Gastroinest Endosc.

[29] Yoo IK, Ko WJ, Kim HS (2020). Anti-reflux mucosectomy using a cap-assisted endoscopic mucosal resection method for refractory gastroesophageal disease: a prospective feasibility study. Surg Endosc.

[30] Sui X, Gao X, Zhang L (2022). Clinical efficacy of endoscopic antireflux mucosectomy vs. Stretta radiofrequency in the treatment of gastroesophageal reflux disease: a retrospective, single-center cohort study. Ann Transl Med.

[31] Benias PC, D'Souza L, Lan G (2018). Initial experience with a novel resection and plication (RAP) method for acid reflux: a pilot study. Endosc Int Open.

[32] Deshmukh A, Parsa N, Elmeligui A, Nieto J (2022). Antireflux band mucosectomy: a novel minimally invasive approach for the treatment of refractory gastroesophageal reflux disease. VideoGIE.

